# CT validation of intraoperative imageless navigation (Naviswiss) for component positioning accuracy in primary total hip arthroplasty in supine patient position: a prospective observational cohort study in a single-surgeon practice

**DOI:** 10.1186/s42836-023-00217-z

**Published:** 2023-12-05

**Authors:** Corey Scholes, Tobias Schwagli, John Ireland

**Affiliations:** 1 EBM Analytics, Sydney, NSW 2065 Australia; 2 Medivation, CH-5200 Brugg, Switzerland; 3 Sydney Bone and Joint Clinic, Sydney, NSW 2560 Australia

**Keywords:** Hip, Arthroplasty, Alignment, Navigation

## Abstract

**Background:**

The aim of this study was to report on the validity of the Naviswiss handheld image-free navigation device for accurate intraoperative measurement of THA component positioning, in comparison with the three-dimensional (3D) reconstruction of computed tomography (CT) images as the gold standard.

**Methods:**

A series of patients presenting to a single-surgeon clinic with end-stage hip osteoarthritis received primary hip arthroplasty with the anterolateral muscle-sparing surgical approach in the supine position. Imageless navigation was applied during the procedure with bone-mounted trackers applied to the greater trochanter and ASIS. Patients underwent routine CT scans before and after surgery and these were analyzed by using three-dimensional reconstruction to generate cup orientation, offset and leg length changes, which were compared to the intraoperative measurements provided by the navigation system. Estimates of agreement between the intraoperative and image-derived measurements were assessed with and without correction for bias and declared cases with potential measurement issues.

**Results:**

The mean difference between intraoperative and postoperative CT measurements was within 2° for angular measurements and 2 mm for leg length. Absolute differences for the two indices were between 5° and 4 mm. Mean bias was 1.9°–3.6° underestimation for cup orientation and up to 2 mm overestimation for leg length change, but absolute thresholds of 10° and 10 mm were not exceeded by 95% limits of agreement (LOA), especially after correction for bias. Four cases (12%) were declared intraoperatively for issues with fixation on the greater trochanter. Inclusion of these cases generated acceptable accuracy overall and their omission failed to improve between-case variability in accuracy or LOA for both offset and leg length.

**Conclusions:**

The accuracy of the Naviswiss system applied during primary THA in a supine position and anterolateral surgical approach falls within clinically acceptable recommendations for acetabular cup placement, femoral offset, and length. With refinements to surgical technique to adapt to the navigation hardware, the system could be further improved with regression-based bias correction.

**Trial registration:**

Registered with the Australian New Zealand Clinical Trials Registry (ACTRN12618000317291)

**Supplementary Information:**

The online version contains supplementary material available at 10.1186/s42836-023-00217-z.

## Introduction

The number of primary total hip arthroplasties (THA) in Australia has risen 125% since 2003, with the revision burden reported to be at 7.6% at the end of 2021 (Australian Orthopaedic Association National Joint Replacement Registry [1]. Accurate alignment of the implant components and limb length equalization during THA is essential for minimizing the risk of revision surgery [19]. Poor positioning of the acetabular cup can cause dislocation, impingement and instability, while a large leg length discrepancy increases the risk of back pain, gait impairment and overall patient dissatisfaction [5, 16, 22, 29]. Leg length discrepancy has also been cited as a major cause of litigation, accounting for 8%–26% of lawsuits following THA [21].

Robotic-assisted THA can improve the accuracy of implant placement and significantly reduce leg length discrepancies [14]. However, the surgical time is prolonged and there are no significant improvements in the rate of complications and implant survivorship [14, 15, 28]. The financial investment and time required to adopt these systems have also prevented their widespread use [23]. Portable navigation systems have been developed to offer a cost-effective, user-friendly and minimally-invasive solution, and have been demonstrated to improve component positioning [26] even when used with different surgical approaches [4, 9, 12].

The Naviswiss (Naviswiss AG, Brugg, Switzerland) is a portable imageless navigation device equipped with an infrared stereo camera and an inertial measurement unit to facilitate implant positioning intraoperatively. The accuracy of the system has been reported as <3° mean absolute error for cup inclination and anteversion when tested in the supine position via an anterolateral THA approach [11], and <3.5° for cup orientation using a direct anterior THA approach with fluoroscopy [20]. However, clinical data for the system has not been reported in Australia, and there is a dearth of information regarding femoral offset and leg length discrepancy.

As such, a trial protocol was developed to assess the accuracy of the Naviswiss system in measuring acetabular cup inclination, acetabular cup version, femoral offset and leg length discrepancy [6]. The aim of this study was to report on the validity of the Naviswiss handheld image-free navigation device for accurate intraoperative measurement of THA component positioning, in comparison with the three-dimensional (3D) reconstruction of computed tomography (CT) images as the gold standard.

## Methods

### Patient selection

The study was embedded within a prospective observational clinical registry, for which ethical approval was obtained from a National Health and Medical Research Council-certified Human Research and Ethics Committee (HREC) (Bellberry; HREC 2017-07-499). The study was also registered with the Australian New Zealand Clinical Trials Registry (ACTRN12618000317291). Adult patients (>18 years) were included in the study if they presented to the participating surgeon with end-stage or rheumatoid arthritis and elected to undergo THA.

Patients who were unable to provide informed consent, or had declined or revoked consent were excluded from the study. Patients were additionally excluded if: they had severe contralateral hip deformity or dysplasia; required a simultaneous bilateral procedure; required an ipsilateral revision procedure; had a short-stem component implanted; were lost to follow-up; use of the navigation system was completely abandoned; received a posterior surgical approach; were revised prior to postoperative imaging being performed; where hardware was unavailable or failed such that intraoperative data could not be retrieved.

### Surgical technique

Patients were administered preoperative antibiotic prophylaxis and were placed in the supine position on a radiolucent table to allow intraoperative imaging of cup and stem positions. Once patients were prepped and draped, the iliac crest was identified and a tracker was fixed prior to the registration of anatomical landmarks. An anterolateral muscle-sparing approach was utilized to expose the hip joint and trochanteric region. A screw with a serrated washer was inserted into the lateral trochanter near the caudal attachment of the gluteus medius, and a stalked tracker was inserted to clear the soft tissues. Registration of the hip joint was then performed. The final intraoperative component positions were logged by the navigation system and transferred by electronic form for further analysis. All surgeries were performed by the senior author.

### Measurement of component positioning

Patients underwent a postoperative CT at the 6-week follow-up, and the data were retrieved and analyzed. The primary study outcomes were extracted for analysis as previously described [6, 25].

- Acetabular cup inclination (ACI)—Angle between the acetabular and longitudinal axes when projected onto the functional pelvic plane (FPP)
- Acetabular cup version (ACV) —Angle between the acetabular axis and the FPP
- Femoral offset (FO) —Difference between the hip centre of rotation (COR) of the operated joint relative to its starting position at the initial assessment on the coronal plane (medial-lateral) within the pelvic coordinate system
- Leg length discrepancy (LLD)—Change in the distance between the greater trochanter tag and the hip COR added to the change in the distance between the centre of the acetabulum and the centre of the cup on the transverse plane (superior-inferior)

CT images were obtained pre- and postoperatively in DICOM (Digital Imaging and Communications in Medicine) format, and information relating to the diagnosis, study or surgery was removed for blinded analysis by an independent researcher.

Inclination and version of the acetabular cup were measured on DICOM files using online software (3D Slicer, www.slicer.org) [7] as previously described [6, 25]. FO and LLD were measured through the assessment of anatomical landmarks picked in pre- and postoperative scans. Coordinate systems for the pelvis and femur were determined based on the anatomic landmarks for the postoperative CT assessments. Parameters were expressed relative to the FPP, with the origin placed at the centre of the line connecting the left and right ASIS. The postoperative position of the cup centre was compared with the native hip COR determined from the preoperative CT. FO and LLD were reported as the pre-to-post change of the femoral coordinate frame relative to pelvis FPP coordinates on the coronal (mediolateral) and transverse (inferior-superior) planes respectively.

### Data and statistical analysis

#### Missing data

Missing data were identified predominantly from intraoperative measurements where technical issues precluded retrieval of certain measurements within a case, but not all (exclusion criterion). Due to the low proportion of missing data <10%, case-wise deletion was performed to restrict analysis on each outcome measure.

#### Reliability

Intraobserver reliability for the image-based measures, including inclination and anteversion relative to the table orientation as well as cup position changes, was assessed using intraclass correlations, with a two-way random effects model on a sample of nine cases. Case identifier and observation (intra-observer) or observer (inter-observer) were considered random effects. The ratings were performed by the same observer on three occasions separated by a minimum of one week, with blinding to previous measures as well as measures by other observers and identified only by case identifier. Standard error of measurement was defined by the root mean square error from the repeated measures analysis of variance [32] with observation or observer as the repeated factor. Standard error of measurement describes the average deviation from one measurement to the next that can be attributed to random observer error.

Interobserver reliability for the image-based measures was derived from the median of the observations from the primary observer as well as singular observations from two additional observers who performed their measurements independently on the same sub-sample (*N *= 9). Intraclass correlation with two-way mixed-effects models was also calculated, and typical error measurement was calculated in the same manner as for interobserver reliability.

#### Agreement and bias correction

The calculation of agreement and bias assessment used the method described in Scholes et al. [25], and is summarized here for clarity. Mean deviation (delta) for each measure was calculated and summary statistics were generated using a bootstrapping approach and repeated with delta converted to absolute values. Bland-Altman plots were generated and limits of agreement were calculated by using a previously published formula [3, 8]. Bias was assessed using linear regression on each measure with adjustments for age, body mass index (BMI) and sex. Bias-corrected estimates of each measure were generated from regression model predictions. Alternative correction for offset and leg length change was performed by dropping values where an intraoperative declaration was made and the summary agreement analysis repeated as described above (Supplementary 1). All statistical analyses were performed using Stata (v17.1, StataCorp, College Station, TX, USA), with alpha set at 5% to indicate significant effects where appropriate.

## Results

### Patient characteristics

**Fig. 1:**
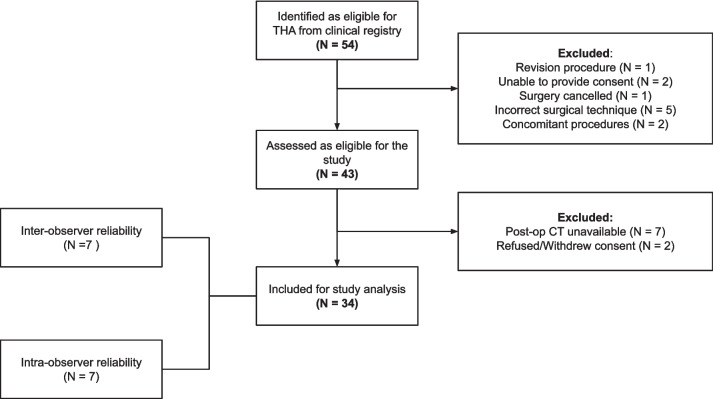
STROBE diagram [31] of patient inclusion into the study analysis

A consecutive sample of 54 primary cases was assessed for eligibility, with 34 included for analysis (Fig. 1). The analysis cohort comprised 42% females, had a mean age of 60.8 years (IQR 50.3–70) and a mean BMI of 29.6 (IQR 27.3–32.4) at surgery.

### Imaging reliability

The imaging analysis demonstrated adequate reliability for both within and between observers for the measures of interest (Tables 1 and 2), with intra-observer standard error of measurement being <1° for cup anteversion and inclination and <1.1 mm for femoral offset and leg length changes (Table 3).

**Table 1:** Individual and average intra-class correlations for intraobserver, image-based measurements

	**Individual**			**Average**		
	**ICC**	**95% LCI**	**95% UCI**	**ICC**	**95% LCI**	**95% UCI**
Inclination_ APP	0.99	0.97	1.00	0.997	0.99	1
Inclination_ FPP	0.986	0.95	1.00	0.995	0.98	1
Version_ APP	0.996	0.986	1.00	0.998	0.99	1
Version_ FPP	0.996	0.987	1.00	0.998	0.996	1
Offset	0.868	0.62	0.97	0.95	0.83	0.99
LLD	0.987	0.955	1.00	0.995	0.984	1

*LCI* lower confidence interval, *UCI* upper confidence interval, *ICC* intra-class correlation, *APP* anatomical pelvic plane, *FPP* functional pelvic plane, *LLD* leg length discrepancy

**Table 2:** Individual and average intra-class correlations for interobserver, image-based measurements

	**Individual**			**Average**		
	**ICC**	**95%LCI**	**95%UCI**	**ICC**	**95%LCI**	**95%UCI**
Inclination_ APP	0.97	0.87	1.000	0.99	0.95	1
Inclination_ FPP	0.96	0.86	0.990	0.987	0.948	1
Version_ APP	0.98	0.94	1.000	0.99	0.978	1
Version_ FPP	0.988	0.959	0.988	0.996	0.986	1
Offset	0.866	0.604	0.973	0.951	0.821	0.99
LLD	0.945	0.823	0.989	0.98	0.933	0.996

*APP*: anatomical pelvic plane, *FPP* functional pelvic plane, *LLD* leg length discrepancy

**Table 3:** Standard error of measurements for image-based analysis of cup position and orientation

	Intraobserver	Interobserver
Inclination_ APP (degree)	0.63	0.82
Inclination_ FPP (degree)	0.57	0.82
Version_ APP (degree)	0.62	0.90
Version_ FPP (degree)	0.55	0.76
Offset (mm)	1.08	1.08
LLD (mm)	0.73	1.05

*APP* anatomical pelvic plane, *FPP* functional pelvic plane, *LLD* leg length discrepancy

### Agreement-intraoperative to imaging

#### Thresholds and declared observations

Intraoperative declarations were made for four patients, with loss of fixation of the tracker on the greater trochanter (*n* = 4). A number of patients were observed to exceed the specified measurement thresholds and had no intraoperative declarations (Table 4). Two patients included in the analysis had an intraoperative declaration recorded. However, their measurements did not exceed the threshold boundaries.

**Table 4:** Patients without an intraoperative declaration that exceeded the measurement thresholds

**Measurement**	**Threshold**	**Units**	**Cases above threshold**	**Proportion above threshold**
Threshold-Inclination	10	degree	2	5.9%
Threshold-Version	10	degree	2	5.9%
Threshold-Offset	10	mm	1	2.9%
Threshold-LLD	10	mm	1	2.9%

**Fig. 2:**
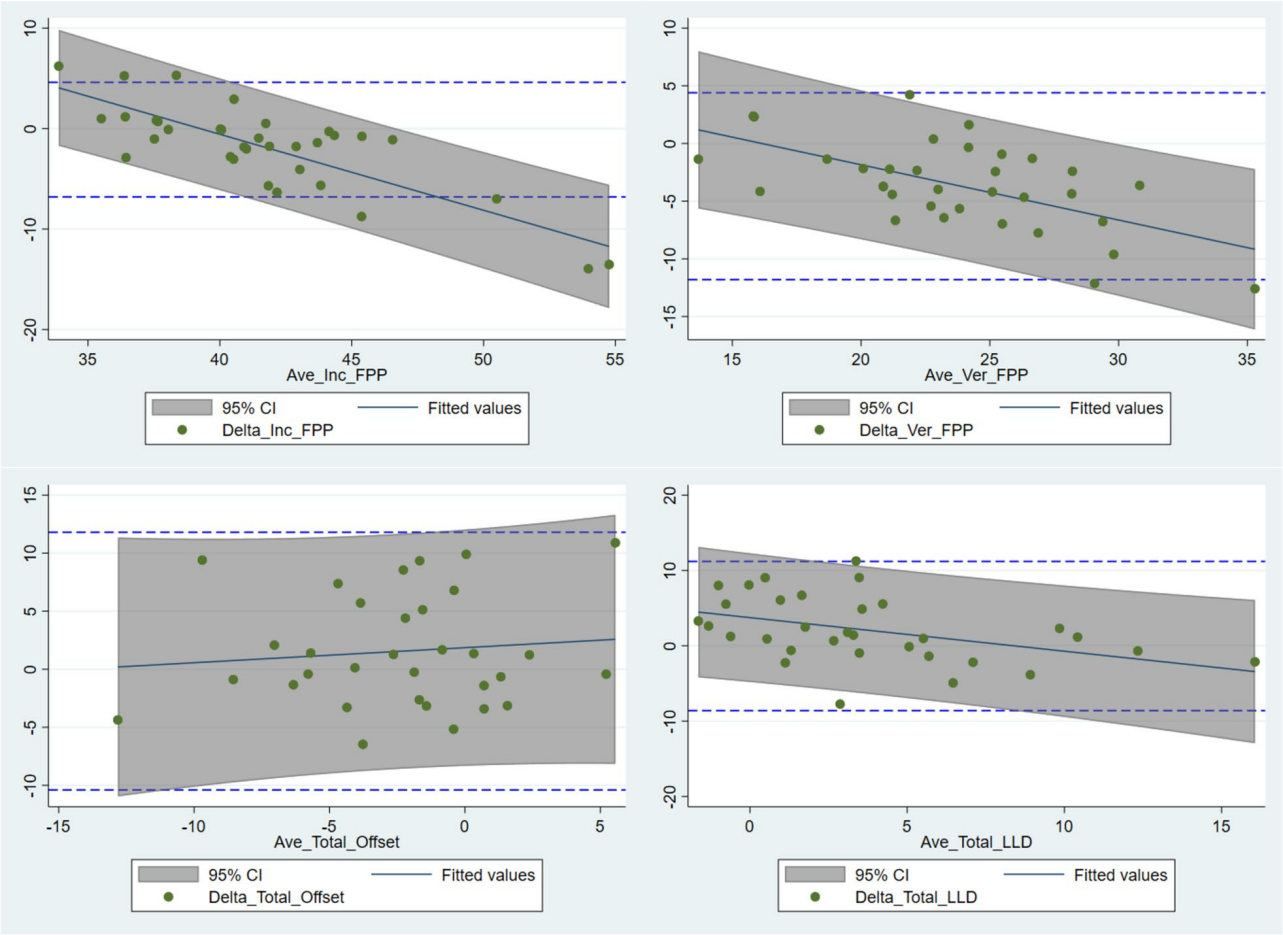
Bland-Altman plots with 95% limits of agreement for inclination, version, offset and leg length. Regression fits with shaded areas denoting 95% prediction intervals indicate the relationship between magnitude and agreement

**Fig. 3:**
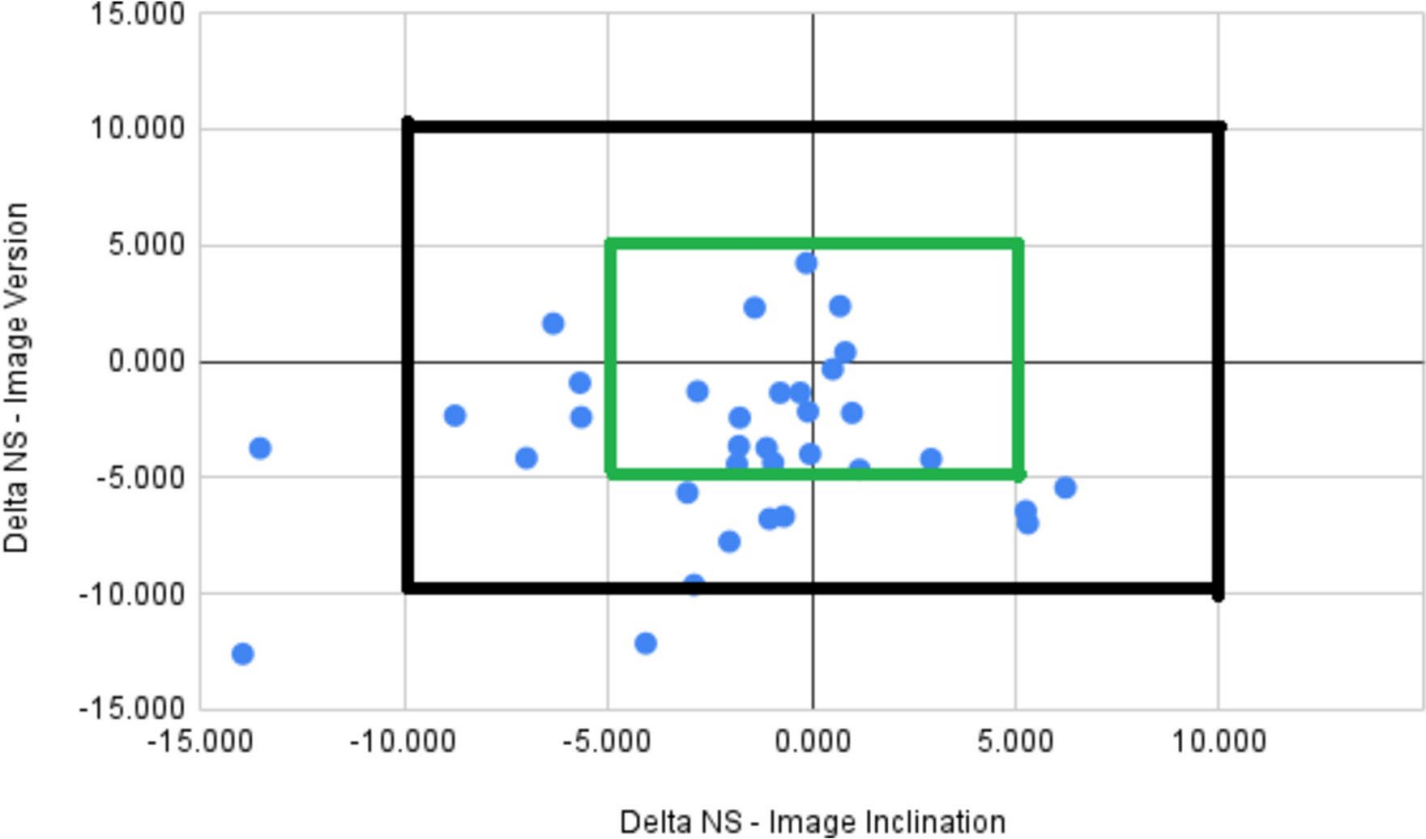
Scatterplot of delta in version vs. inclination for all cases. Outer box -10° threshold; Inner box -5° threshold

The mean delta (bias) between the intraoperative measurements and the postoperative imaging are summarized in (Table 5). The 95% limits of agreement for uncorrected data exceeded 10 degrees for inclination and version, and 10 mm for offset and LLD respectively (Table 5, Fig. 2). The linear fit of the average to the delta indicated that the bias between the navigation and the CT measurements was not constant across magnitude for inclination, version and offset (Fig. 2, Table 6). Overall, 91.2% of cases (95%CI 76.3–98.1) were within 10° of the image-measured measurements for both inclination and version (Fig. 3).

**Table 5:** Summary of mean differences between intraoperative and image-based measurements. *P*-value is relative to the mean delta being different from zero

**Sign**	**Mean Delta**	**SE**	**SD**	**95%LCI**	**95%UCI**	***P*-value**	**Lower LOA**	**Upper LOA**
Inclination	-1.9	0.77	4.5	-3.4	0.4	0.015	-10.7	6.9
Version	-3.6	0.63	3.7	-4.8	-2.4	<0.001	-10.8	3.6
Offset	1.5	0.85	5.0	-0.1	3.2	0.069	-8.2	11.2
Leg Length	2.1	0.76	4.4	0.5	3.6	0.009	-6.6	10.8
**Absolute**								
Inclination	3.3	0.61	3.6	2.1	4.5			
Version	4.3	0.51	3.0	3.3	5.2			
Offset	3.9	0.56	3.3	2.8	5			
Leg Length	3.7	0.55	3.2	2.7	4.8			

*LOA* limits of agreement, *LCI*: lower confidence interval, *UCI* upper confidence interval, *SE* standard error of the estimate, *SD* standard deviation

**Table 6:** Linear fit of average of measurements to delta of measurements

	**Coefficient**	**SE**	**95%LCI**	**95%UCI**	***P*-value**	**Adjusted R-sq**
Inclination	-0.76	0.12	-1.00	-0.51	<0.001	0.65
Version	-0.48	0.12	-0.71	-0.25	<0.001	0.35
Offset	0.13	0.26	0.38	0.64	0.617	-0.02
Leg Length	-0.45	0.14	-0.72	-0.17	0.001	0.18

*LCI* lower confidence interval, *UCI* upper confidence interval, *SE* standard error of the estimate

### Factors associated with agreement and bias correction

The regression results indicated a significant magnitude-dependent bias for inclination (*P* = 0.014) and offset (*P* = 0.002) (Supplementary 2). Bias correction applied to the intraoperative measures removed overall bias and shrank the between-case variation (SD) of delta by 1%–20%, and by 22%–41% for absolute values (Table 7). Bias correction also shrank the mean absolute delta by 5%–35% relative to the uncorrected values. Conversely, by omitting declared observations for offset and leg length (Table 8), mean absolute error was not reduced and between-case variability increased by 5%–7%.

**Table 7:** Summary of mean differences between intraoperative and image-based measurements for bias corrected intraoperative measures

**Sign**	**Mean Delta**	**SE**	**SD**	**95%LCI**	**95%UCI**	**Lower LOA**	**Upper LOA**
Inclination	-0.002	0.63	3.7	-1.2	1.2	-7.2	7.2
Version	0	0.59	3.4	-1.2	1.2	-6.7	6.7
Offset	-0.003	0.68	4.0	-1.3	1.3	-7.8	7.8
Leg Length	0.006	0.75	4.4	-1.5	1.5	-8.6	8.6
**Absolute**							
Inclination	3	0.36	2.1	2.2	3.7		
Version	2.8	0.37	2.2	2	3.5		
Offset	3.3	0.35	2.0	2.6	4		
Leg Length	3.5	0.43	2.5	2.7	4.3		

*LOA* limits of agreement, *LCI* lower confidence interval, *UCI* upper confidence interval, *SE* standard error of the estimate, *SD* standard deviation

**Table 8:** Summary of mean differences between intraoperative and image-based measurements with declaration cases omitted (*N* = 30)

**Sign**	**Mean Delta**	**SE**	**SD**	**95% LCI**	**95% UCI**	***P*-value**	**Lower LOA**	**Upper LOA**
Offset	1.3	0.88	5.1	-0.4	3	0.146	-8.8	11.4
Leg Length	2.4	0.78	4.5	0.9	3.9	0.002	-6.5	11.3
**Absolute**								
Offset	3.8	0.55	3.2	2.7	4.8			
Leg Length	3.8	0.59	3.4	2.6	4.9			

*LOA* limits of agreement, *LCI* lower confidence interval, *UCI* upper confidence interval, *SE* standard error of the estimate, *SD* standard deviation

## Discussion

The aim of this study was to report on the validity of an imageless navigation system (Naviswiss) for intraoperative measurement of THA component, in comparison with the three-dimensional (3D) reconstruction of computed tomography (CT) images as gold standard. The results identified reasonable accuracy for the metrics of interest with opportunities for further development identified.

The mean absolute deviation of acetabular inclination (3.3, 95%CI 2.1–4.5) between the navigation system and the CT-based analysis was comparable to the deviation reported by Hasegawa et al. in .2022 (2.8, 2.3–3.3) in the supine position, and by Scholes et al. [25] in the lateral decubitus position (3.6, 2.6–4.7), but it was higher than the pooled deviation (2.6, 2.4–2.8) for previous studies in the supine patient position using CT-based, imageless and accelerometry systems (Supplementary 3). In contrast, anteversion mean absolute deviation (4.3, 3.3–5.2) was greater than that reported by Hasegawa (2.8, 2.3–3.3) but comparable to pooled deviation for previous studies (3.6, 3.4–3.8). Overall, the mean bias was 1.9°–3.6° underestimation for cup orientation and up to 4 mm overestimation for leg length change, with 95% LOA at or below 11° for orientation and 6-11 mm for offset/leg length change. Absolute thresholds of 10° and 10 mm were established *a priori* in the study protocol [6] and were exceeded by 95% LOA, but this was reduced by bias correction to <10. Between-patient variation in published guidelines for cup orientation varies between 5 and 12° for inclination and up to 18° for version [10]. In general, less than 10 mm of LLD is considered acceptable after THA [18]*.* In addition, a simulation study [27] reported impingement and loss of motion range with a 4 mm medialization/lateralization of the cup, although this amount of change was not justified in their methods. The bias-corrected LOA in the present study suggests that 95% of the patient sample would fall within these tolerances for cup orientation, but not for offset. The findings are comparable to those reported by Scholes et al. in [25], who performed the same analysis with patients in the lateral decubitus position. A better understanding of the factors contributing to error in patients, the extremes of anthropometry and morphology, irrespective of surgical approach or patient position, may provide an important direction for further research.

In any validation study, a desire to explain deviations from true agreement is a natural progression of the analysis. In this study, a biphasic pattern of magnitude-dependent bias was observed for inclination (FPP), version and leg length change, and was also noted by Scholes et al. in [25]. The navigation system tended to overestimate smaller average measurements and underestimate larger averages (Fig. 2). While bias correction was able to re-centre the sample around zero and reduce between-patient variation, further work is needed to validate regression-based bias correction algorithms to mitigate the magnitude-dependency (slope) [25]. In addition, further work may be required to improve tracker fixation for offset and leg length with 4 cases (11.8%) declared intraoperatively for issues with fixation on the greater trochanter. In contrast to the study by Scholes et al. [25], the inclusion of these cases generated acceptable accuracy overall, while their omission worsened the between-case variability in accuracy and increased the LOA for both offset and leg length. A previous study [11], mentioned the potential vulnerability of the system to pin fixation on the iliac crest.

In the context of the magnitude-dependent bias of the system, the most realistic explanation for deviations from true agreement is a combination of imaging measurement error (all measures), dampening of the intraoperative measurements from a combination of soft tissue coverage and draping across key anatomical landmarks, as well as soft-tissue interference due to the proximity of the surgical incision (offset, leg length). Three-dimensional CT-based measurements are considered the gold standard when determining acetabular cup position and leg length postoperatively [2, 13, 24]. The present study, however, demonstrated lower reliability of the inter-observer imaging analysis with up to 1° for cup anteversion to 1.1 mm for LLD. In CT-based texture analysis, it has been demonstrated that the robustness, repeatability and reproducibility of measurements are sensitive to the scanner and scanning parameters [30]. The impact of radiological environment and context on postoperative measurements in THA has not been widely explored, but cannot be disregarded when comparing the results of different investigators and investigations. In addition, the selection of manual landmarks on CT datasets, while considered reliable, can still result in THA acetabular abduction and anteversion angle between-case variance of up to 2.5° [17]. In this study, the combination of scans performed on different scanners (patient access and convenience) and intra/inter-observer error may have contributed to the typical error of the imaging measurements. For example, the typical error for offset measurement was equal to 29% of the navigation absolute error. The present results, as well as others that have relied on CT/radiographic validation, should be interpreted with these findings in mind.

While the use of imageless navigation in the supine patient position is not as vulnerable to position changes as the lateral decubitus position [25], many of the issues raised by Scholes et al. [25] also apply to validation attempts in the supine position. Certainly, the mean BMI of the present sample is comparable to their study, while general approaches to draping and soft tissue distribution across key landmarks are almost identical. The replication of magnitude-dependent bias, as well as improved overall accuracy through bias correction in both studies suggests that intraoperative accuracy could be further improved by targeting potential measurement dampening. Anecdotally, these issues are exacerbated with increase in patient size and the amount of soft tissues distributed around the surgical field. A potential for interference between the soft tissue and the tracker fixed to the greater trochanter may be somewhat specific to the present study, however, due to the location of the anterolateral surgical incision. The incision created a segment of tissue flap that had a propensity to physically interact with the trochanter tracker without mitigation strategies in place. When higher BMI patients co-existed with a greater propensity for soft tissue distribution around this area, the tissue flap could cause unpredictable deviations in measurement through the course of the procedure. Of note were the attempts at mitigation that were implemented over the course of the trial by adjusting the incision location to reduce the amount of loose tissues, as well as taking additional steps to secure the flap and prevent abutment against the tracker itself. Further work is required to quantify the efficacy of these strategies.

The present study established that the system of interest was capable of providing comparable accuracy to similar systems in a broad range of patient populations, while identifying potential avenues of further development to achieve superior accuracy. Nevertheless, the findings and interpretations should be considered in light of the study’s limitations. Firstly, as discussed, variability in the imaging situation from one patient to another may have contributed to some of the errors observed in the imaging analysis. Clinical imaging in our region is referral-based, for the patient to organize a booking with a provider based on convenience and usually geographic location relative to their residence. While every attempt to standardize the imaging protocol was made with usual providers, some patients undertook their imaging studies outside this network. Despite the mitigation attempts, there is still the potential for variable imaging parameters due to different machines being used. Secondly, comparisons with the related literature remain problematic, and according to Scholes et al. [25], there are a range of different analytical techniques that have been used to describe the validity of the system intraoperatively, across a broad range of populations. However, the most problematic is the metric(s) used to report accuracy, which tend to use a summary of average deviation. Considering the findings of the present study, the assumption that error does not vary based on measurement magnitude clearly does not hold, although it is impossible to determine if this phenomenon has been observed elsewhere, as no other studies have employed Bland-Altman plots or similar techniques to describe this relationship. This makes an average deviation inappropriate for the description of average error and future work should report use of regression-based metrics. Thirdly, while this study did not represent the learning curve for the operating surgeon, the early part of the present series was characterized by the continued development of the system with the manufacturer, with a limited number of cases having to be abandoned entirely due to technical faults (e.g., system lockup, dropped tracker tags). This may have inserted a low level of selection bias into the series and maybe a consideration for clinical interpretation for those less experienced with imageless navigation in THA. Lastly, the current series was deliberately restricted to primary, relatively uncomplicated THA, which might limit its generalizability to more extreme morphological presentations (revision, tumor) where navigation may be of specific benefit. Further work is required to extend the validity of the system to this broader case mix.

## Conclusion

The navigation system assessed in a primary THA cohort for end-stage hip osteoarthritis provided acceptable validity within clinical recommendations for cup orientation, femoral offset and leg length in the supine patient position. With additional attention to the proposed mechanisms for error identified in the present analysis, the application of the imageless system of interest to pathological anatomy in the context of challenging anthropometry, varying patient position and surgical approach, could become the standard for improving surgical outcomes in primary THA.

### Supplementary Information

**Additional file 1:** **Supplementary material 1.** Code

** Additional file 2:** **Supplementary material 2.** Regression model results summary

** Additional file 3:** **Supplementary material 3.** Summary of validation findings

## Data Availability

Upon reasonable request to the authors, only de-identified data (i.e., data with sensitive and personal information removed), will be provided as per the ANDS Publishing and Sharing Sensitive Data Guide.
