# Supplementary material for: CT validation of intraoperative imageless navigation (Naviswiss) for component positioning accuracy in primary total hip arthroplasty in supine patient position: a prospective observational cohort study in a single-surgeon practice
Source: Arthroplasty. 2023 Dec 5;5:63. doi: 10.1186/s42836-023-00217-z (PMC10696686; doi:10.1186/s42836-023-00217-z)
Supplement: Supplementary file 2 — Additional file 2: Supplementary material 2. Regression model results summary [file 42836_2023_217_MOESM2_ESM.pdf]

Supplementary material 2 - Regression Models not used

Table 1: Regression summary delta inclination (FPP)

Linear regression		
Number of obs	=	34
Replications	=	100
Wald chi2(4)	=	7.25
Prob > chi2	=	0.1233
R-squared	=	0.2994
Adj R-squared	=	0.2028
Root MSE	=	3.9841

delta_in~fpp	Observed coefficient	Bootstrap std. err.	z	P>|z|	Normal-based [95% conf.	interval]
ns_inc	-.7202958	.2922558	-2.46	0.014	-1.293107	-.147485
bmi	-.2253103	.1315003	-1.71	0.087	-.4830463	.0324256
ageatsurgery	-.0356231	.0797826	-0.45	0.655	-.1919941	.1207479
sexcode						
Male	.8781924	1.416129	0.62	0.535	-1.89737	3.653755
_cons	36.44397	15.52767	2.35	0.019	6.01029	66.87765

Table 2: Regression summary delta version (FPP)

Linear regression		
Number of obs	=	34
Replications	=	100
Wald chi2(4)	=	4.68
Prob > chi2	=	0.3222
R-squared	=	0.1154
Adj R-squared	=	-0.0066
Root MSE	=	3.7988

delta_ve~fpp	Observed coefficient	Bootstrap std. err.	z	P>|z|	Normal-based [95% conf.	interval]
ns_ver	-.2725811	.1661733	-1.64	0.101	-.5982748	.0531126
bmi	-.1300883	.1714082	-0.76	0.448	-.4660422	.2058656
ageatsurgery	.0334747	.0745442	0.45	0.653	-.1126293	.1795787
sexcode						
Male	1.628467	1.44538	1.13	0.260	-1.204427	4.461361
_cons	2.665045	7.352848	0.36	0.717	-11.74627	17.07636

Table 3: Regression summary delta total offset

Linear regression

Number of obs	=	32
Replications	=	100
Wald chi2(4)	=	13.56
Prob > chi2	=	0.0088
R-squared	=	0.3512
Adj R-squared	=	0.2550
Root MSE	=	4.1696

delta_tota~t	Observed coefficient	Bootstrap std. err.	z	P>|z|	Normal-based [95% conf.	interval]
ns_offset	.569177	.1869792	3.04	0.002	.2027044	.9356496
bmi	.0089397	.2717262	0.03	0.974	-.5236338	.5415132
ageatsurgery	-.0563344	.0954872	-0.59	0.555	-.2434859	.130817
sexcode						
Male	.865698	2.410951	0.36	0.720	-3.859678	5.591074
_cons	5.854657	12.20774	0.48	0.632	-18.07207	29.78138

Table 4: Regression summary delta leg length difference

Linear regression

Number of obs	=	32
Replications	=	100
Wald chi2(4)	=	0.84
Prob > chi2	=	0.9328
R-squared	=	0.0439
Adj R-squared	=	-0.0977
Root MSE	=	4.6307

delta_tota~d	Observed coefficient	Bootstrap std. err.	z	P>|z|	Normal-based [95% conf.	interval]
ns_lld	.1194275	.2612432	0.46	0.648	-.3925997	.6314547
bmi	-.1118321	.2165626	-0.52	0.606	-.536287	.3126228
ageatsurgery	.0281283	.1291462	0.22	0.828	-.2249936	.2812501
sexcode						
Male	1.293895	1.850367	0.70	0.484	-2.332758	4.920548
_cons	1.841925	11.61245	0.16	0.874	-20.91806	24.60191
